# Mature Teratoma of the Cerebellum with Formed Extracranial Component

**DOI:** 10.3390/jcm14061994

**Published:** 2025-03-15

**Authors:** Agnieszka Nowacka, Ewa Ziółkowska, Wojciech Smuczyński, Dominika Bożiłow, Maciej Śniegocki

**Affiliations:** 1 Department of Neurosurgery, Collegium Medicum in Bydgoszcz, Nicolas Copernicus University in Toruń, ul. Curie Skłodowskiej 9, 85-094 Bydgoszcz, Poland; 2 Department of Pediatrics, Washington University School of Medicine, St. Louis, MO 63110, USA; 3 Department of Physiotherapy, Collegium Medicum in Bydgoszcz, Nicolas Copernicus University in Toruń, ul. Techników 3, 85-801 Bydgoszcz, Poland; 4 Anaesthesiology and Intensive Care Clinical Ward, The 10th Military Research Hospital and Polyclinic, ul. Powstańców Warszawy 5, 85-681 Bydgoszcz, Poland

**Keywords:** teratoma, mature teratoma, CNS tumors, GCT tumors, GCT, germ cell tumors, CNS germ cell tumors, intracranial teratoma, cerebellum teratoma, cerebellum tumors

## Abstract

**Background:** Intracranial teratomas are very rare in adults, representing only 0.3–0.5% of all primary brain tumors. They originate from all three germ layers, and are classified as mature, immature, or malignant. Mature teratomas constitute the most prevalent type in the adult population, commonly originating from midline structures such as the pineal and suprasellar regions. However, the localization of these tumors within the cerebellum is exceedingly rare, with only a limited number of cases reported globally. In this manuscript, we describe, to the best of our knowledge, the first documented case of a young adult patient presenting with a mature teratoma situated between the cerebellar hemispheres. Notably, this tumor was accompanied by occipital bone loss, through which a tumor pedicle extended, forming an extracranial component. **Methods**: After analyzing the clinical picture and additional examinations, the patient was classified for surgery. The intracranial part of the tumor contained numerous cysts with yellow fluid, a tooth, and fat tissue. The tumor was removed radically, with its extracranial part. **Results**: On the fourth day after surgery, the patient was discharged from the clinic in a good general condition, walking, with marked cerebellar symptoms. In a follow-up at 6 months postoperatively, the neurological examination was normal, with no headaches. MRI at the 6 months follow-up did not show any residual or recurrent tumor. **Conclusions:** Histopathological examination confirmed the diagnosis of mature teratoma.

## 1. Introduction

Intracranial teratomas in adults are exceedingly rare, accounting for only 0.3–0.5% of all primary brain tumors [1]. These tumors are composed of tissues from all three germ layers: ectoderm, mesoderm, and endoderm [2]. Histologically, they are classified into mature, immature, and teratomas with malignant transformation [3]. Mature teratomas are the most common type found in adults, often presenting in midline structures, such as the pineal and suprasellar regions [4]. Their clinical presentation varies depending on their size and location, ranging from headaches and seizures to focal neurological deficits [1,3]. Complete surgical resection is the treatment of choice, and usually results in a favorable prognosis [4,5].

The location of a teratoma in the cerebellum in adults is extremely rare—so far, only few cases have been reported in the world (Table 1). In this paper we present, to the best of our knowledge, the first case of a young adult with a mature teratoma situated between the cerebellar hemispheres, accompanied by occipital bone loss, through which a tumor pedicle extended, forming its extracranial component.

## 2. Case Report

**Figure 1:**
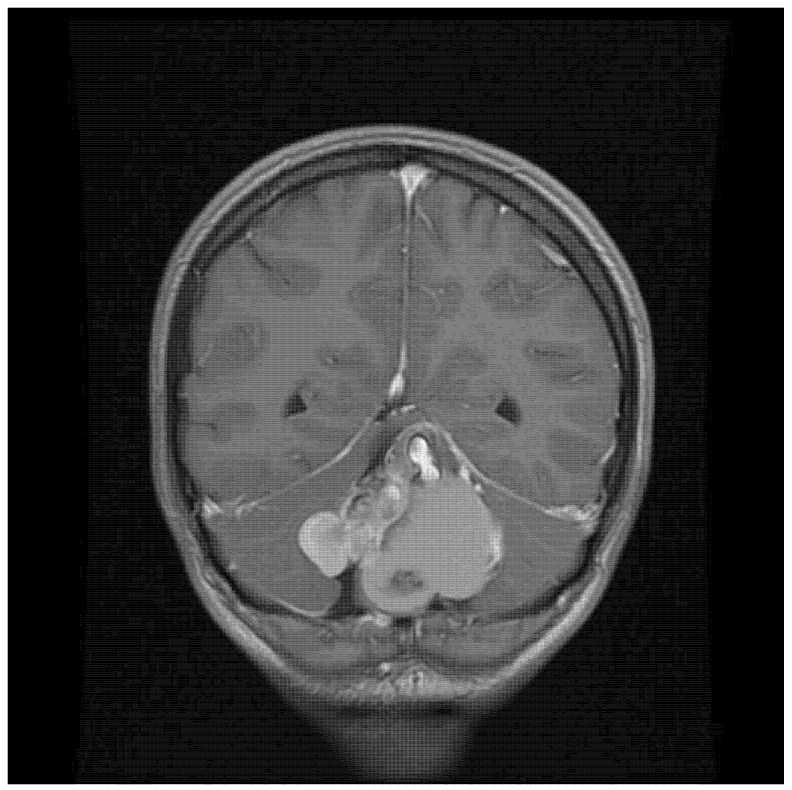
A coronal contrast-enhanced T1-weighted image showing the tumor located between the cerebral hemispheres, with a visible tooth.

**Figure 2:**
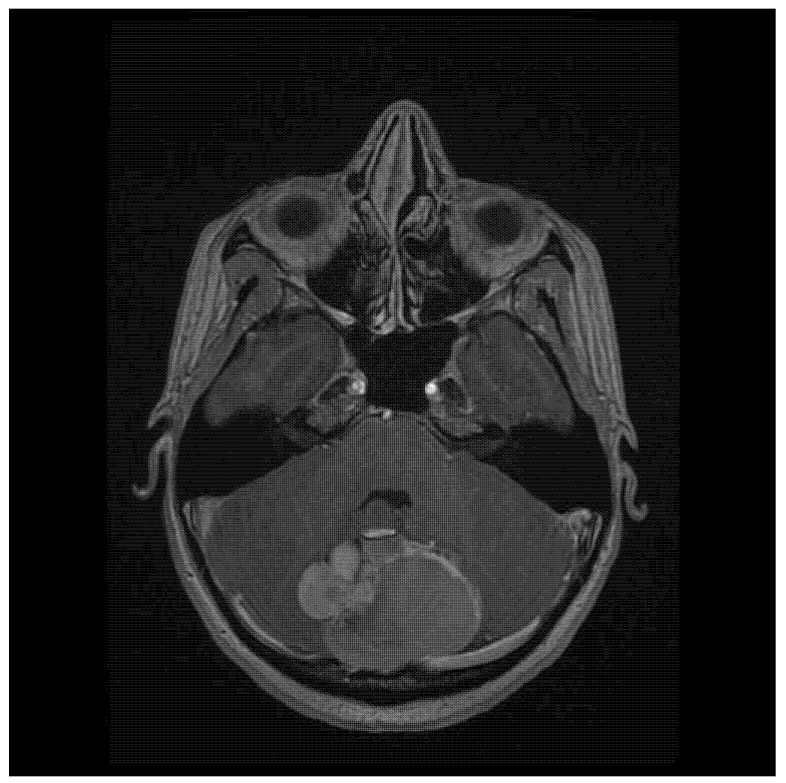
An axial contrast-enhanced T1-weighted image showing the tumor and the bone loss of the occipital bone.

**Figure 3:**
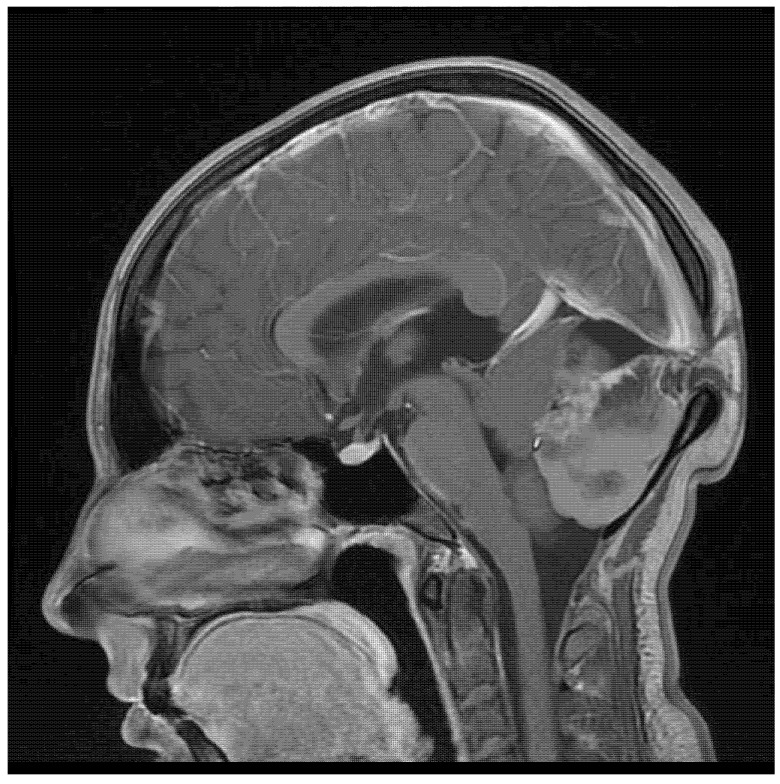
A sagittal contrast-enhanced T1-weighted image showing the tumor with bone loss, through which a tumor peduncle is passing, forming its extracranial part.

A 22-year-old woman presented with severe headache (pain level 7/8 on a Visual Analogue Scale—VAS) that had developed over the previous couple of days, which she described as the worst one she had experienced in her life (pain level VAS 10) on the day of hospital admission. There was no history of chronic diseases. The patient reported a palpable bulge in the occipital region, which she had had since birth. Physical examination on admission produced the following findings: 15 points on the Glasgow Coma Scale (GCS), and no neurological deficits (no paresis, no meningeal and cerebellar symptoms, walking independently). Cranial MRI (magnetic resonance imaging) showed a pathological mass located between the cerebral hemispheres (57 × 40 × 52 mm), with a heterogeneous signal suggesting calcified elements and a heterogeneous contrast area in the middle and right (Figure 1). At the height of the lesion, the occipital bone had an 8.5 mm bone loss in the midline (Figure 2), with a low-signal 3.8 mm wide stride present within it, running from the described mass and ending in subcutaneous adipose tissue (Figure 3).

After analyzing the clinical picture and additional examinations, the patient was classified for surgery. The tumor was resected through a midline suboccipital approach in a seated position. Bone loss was revealed, through which the tumor peduncle was passing, forming the extracranial part, which contained hair strands. Craniotomy under the bone loss was performed. The intracranial part of the tumor contained numerous cysts with yellow fluid, a tooth, and fat tissue. The tumor was removed radically, with its extracranial part. Histopathological examination confirmed the diagnosis of mature teratoma. On the fourth day after surgery, the patient was discharged from the clinic in a good general condition, walking, with marked cerebellar symptoms. In a follow-up at 6 months postoperatively, the neurological examination was normal, with no headaches. MRI at the 6 months follow-up did not show any residual or recurrent tumor.

## 3. Discussion

Intracranial teratomas, arising from germ cells within the brain, are histopathologically classified into mature, immature, and malignant types, each with distinct characteristics and prognoses [19].

- Mature teratomas are typically benign tumors composed of well-differentiated tissues from all three germ layers (ectoderm, mesoderm, and endoderm), with low mitotic activity, without necrosis [2,20,21]. While they can occur in various locations within the central nervous system, they are often found in midline structures, such as the pineal and suprasellar regions [2]. Complete surgical resection, as the preferred treatment for mature teratomas, is usually associated with a favorable prognosis [22] [2,20].
- Immature teratomas are characterized by the presence of undifferentiated or embryonic tissues, often with neuroectodermal components [21,23,24,25]. This presence of immature tissue signifies incomplete differentiation, and is associated with a higher risk of malignant transformation and, subsequently, poorer outcomes compared to mature teratomas [23,24,25]. Immature teratomas exhibit rapid growth and can lead to substantial complications, especially in cases involving infants [26].
- Teratomas with somatic-type malignancy exhibit aggressive characteristics, including rapid growth and the potential for metastasis [27]. They can arise de novo or from pre-existing immature teratomas that undergo malignant transformation, which most commonly manifests as rhabdomyosarcoma or an undifferentiated sarcoma, and, less frequently, as squamous cell carcinoma or adenocarcinoma [21,27,28]. The presence of yolk sac tumor elements can also give rise to enteric-type adenocarcinoma [21]. Immunohistochemistry plays a crucial role in diagnosing malignant teratomas, differentiating the malignant components from the benign tissues within the teratoma [21]. Markers such as vimentin, desmin, smooth muscle actin, S-100, CD99, and glial fibrillary acidic protein are used to identify sarcomatous transformation, while cytokeratins (CK20, CK7) and p53 are helpful in cases of carcinomatous transformation [21]. The presence of these markers, along with the histologic appearance, confirms the diagnosis and guides a multimodal treatment approach involving surgery, chemotherapy, and radiation therapy [21,27,29].

Intracranial teratomas can present with various abnormal structures, reflecting their heterogeneous composition derived from all three germ layers [30]. Rudimentary or partially formed organ structures, such as optic vesicles containing immature neuroectodermal tissue, have been reported, although they are exceptionally rare [24]. The presence of primitive neuroectodermal tissue within immature teratomas raises concerns of potential malignant transformation [28]. More commonly, these tumors exhibit a diverse mix of tissues, including cartilage, bone, muscle, hair, and glandular structures, contributing to their complex and often unpredictable clinical course [30,31].

The location of an intracranial teratoma significantly influences its clinical presentation, management, and prognosis. These tumors frequently arise along midline structures, particularly in the pineal and suprasellar regions, due to their origin from primordial germ cells [2,32]. Teratomas can also develop within the ventricular system, often presenting with signs of increased intracranial pressure due to obstructing cerebrospinal fluid flow, and causing hydrocephalus [2,32]. While being exceptionally rare in adulthood, mature teratomas can occur in the posterior fossa, sometimes mimicking other conditions like intracranial hemorrhage on imaging, due to their heterogeneous composition [17,33]. The tumor’s location often dictates the presenting symptoms. Pineal region teratomas may cause Parinaud’s syndrome (impaired upward gaze) for the region involved and hydrocephalus, while suprasellar tumors can cause visual impairment, and, when extending into the pituitary fossa, can cause symptoms like visual disturbances, diabetes insipidus, and hypopituitarism [34,35,36]. Posterior fossa teratomas can cause cerebellar dysfunction, cranial nerve palsies, and obstructive hydrocephalus, presenting symptoms like headaches, nausea, and vomiting [33]. Understanding the typical locations and associated clinical presentations of intracranial teratomas is crucial for prompt diagnosis and appropriate management.

Magnetic resonance imaging (MRI) is essential for diagnosis, management, prognostic assessment, and determining the need for adjuvant therapies for intracranial teratomas. MRI reveals the tumor’s location, size, and heterogeneous composition, which is crucial for surgical planning and prognostication. These tumors typically exhibit heterogeneous signal intensity on T1- and T2-weighted images, reflecting their diverse tissue components, including fat, calcifications, and cystic areas [30,37]. The heterogeneous appearance is further highlighted by contrast enhancement, although atypical homogeneous enhancement can occur, which may indicate a less aggressive tumor behavior [30]. Advanced MRI techniques, such as magnetic resonance spectroscopy and diffusion-weighted imaging, can further characterize the tumor and aid in differentiating it from other intracranial lesions [38]. MRS (magnetic resonance spectroscopy) can identify specific metabolites within the tumor, while fMRI (functional magnetic resonance imaging) can provide additional insights into its functional status [38]. Integrating MRI with other imaging modalities, such as computed tomography (CT) and positron emission tomography (PET), can provide a comprehensive evaluation of the tumor’s extent and metabolic activity [38]. CT is particularly useful for visualizing calcifications and bony involvement, while PET can assess the tumor’s metabolic rate, which can be helpful in differentiating benign from malignant teratomas [38]. Overall, detailed preoperative imaging is essential for planning the surgical resection of intracranial teratomas [2,39]. It allows for precise localization of the tumor, defining its relationship to critical neurovascular structures and assessing the degree of involvement with surrounding brain tissue. This information is crucial for selecting the optimal surgical approach, minimizing the risk of complications, and maximizing the chances of complete resection, which is the primary goal for achieving a favorable outcome, especially in mature teratomas [2,39]. In complex cases, where the tumor involves eloquent brain regions or major blood vessels, advanced imaging techniques like fMRI and magnetic resonance angiography can further aid surgical planning by providing functional and vascular mapping [2]. This detailed imaging roadmap helps surgeons to anticipate challenges, tailor their approach, and minimize potential morbidity.

Measuring beta-human chorionic gonadotropin (β-hCG) and alpha-fetoprotein (AFP) levels in both cerebrospinal fluid and serum is a valuable diagnostic tool for intracranial teratomas [21,40,41,42]. Interestingly, β-hCG concentrations tend to be significantly higher in CSF, while AFP levels are slightly higher in serum [21,40]. Therefore, analyzing both serum and CSF provides a more complete picture [21,40]. The absence of elevated AFP and β-hCG can help to rule out other GCTs (germ cell tumors) and guide treatment decisions [21,40,41]. Additionally, CSF cytology is crucial for detecting tumor dissemination into the CSF, which is associated with a poorer prognosis [21,40]. Mature teratomas have also occasionally demonstrated elevated levels of additional biomarkers, such as CA19-9 (carbohydrate antigen) [41].

Surgical intervention is the cornerstone of intracranial teratoma management. The primary goal is gross total resection, which offers the best chance for a cure, and reduces recurrence risk [2,5,21,22,29]. However, achieving complete resection can be challenging due to the tumor’s location, often nestled near vital structures within the brain [2,11,22]. Several surgical approaches exist, each with its own benefits and drawbacks. Traditional craniotomy provides direct access to the tumor, facilitating resection, but carries a higher risk of complications [25,43]. Minimally invasive endoscopic approaches, such as the endonasal route, offer reduced surgical trauma and faster recovery, particularly for tumors located in the suprasellar region [29,35,39]. The choice of surgical technique depends on factors like tumor location, size, and the surgeon’s experience. Intraoperative complications, such as bleeding, can occur, especially with immature teratomas, which tend to be more vascular [25,44]. Postoperative outcomes are generally positive, with most patients experiencing significant symptom relief and no recurrence on follow-up imaging; however, postoperative complications can include neurological deficits or hydrocephalus, depending on the affected brain area, and may necessitate further surgical interventions [20,22].

The overall prognosis varies depending on the tumor type. Mature teratomas with complete resection generally have a better prognosis (10-year survival rate—more than 90%) after surgery than immature (5-year survival rate—around 70%) or malignant teratomas, due to their invasive nature, which may require adjuvant therapies like chemo- or radiotherapy to control residual disease or prevent recurrence [2,3,5,8,11,21,25,27,29,34,43,44,45,46]. The rarity of these tumors poses challenges in establishing standardized treatment protocols, and the potential for severe complications, particularly in congenital cases, underscores the need for a multidisciplinary approach. Further research into genomic characterization may pave the way for targeted therapies, potentially improving outcomes for patients with malignant or recurrent intracranial teratomas.

## Figures and Tables

**Table 1:** Mature teratoma in cerebellar region.

Age/Sex	Location	Histology	Symptoms	References
50/male	vermis and left hemisphere	mature teratoma	nausea, vomiting, headache, dizziness	[6]
47/female	left cerebellar hemisphere, apart from midline	mature teratoma	headache, dizziness, and nausea	[7]
66/male	vermis	mature teratoma	chronic occipital headache, episodes of severe vertigo	[8]
42/female	vermis and left hemisphere	mature teratoma	vomiting, progressive headache	[9]
41/female	cisterna magna	mature teratoma	right-sided sudden hearing loss, vertigo, dizziness	[10]
70/female	cerebellopontine angle	mature teratoma	headache, vomiting, gait disturbance, right-sided ataxia	[11]
28/male	whole posterior fossa, arising from roof of fourth ventricle	mature teratoma	occipital headache	[12]
19/female	supramedial cerebellum (dorsal side of midbrain and upper pons)	mature teratoma	intractable yawning	[13]
59/male	cerebellum—midline	mature teratoma	evaluation for metastases	[14]
65/male	vermis, paravermis, and in fourth ventricle	mature teratoma	intense headache, nausea, vomiting, gait ataxia, orizontal nistagmus, dismetria, disdiadocokinezia—predominant on left side, long tract signs—predominant on left side	[15]
24/female	right cerebellopontine angle	mature teratoma	cerebellar ataxia, sensorineural hearing loss, decreased palatal movements and facial sensations, nystagmus	[16]
60/male	vermis	mature teraroma	dysarthria, left hemiparesis	[17]
26/female	cisterna magna	mature teratoma	vomiting, headache, vertigo	[3]
41/male	vermis	mature teratoma	headache, coma	[18]
22/female	cerebellum—midline	mature teratoma	severe headache	current case

## Data Availability

No new data were created or analyzed in this study. Data sharing is not applicable to this article.
